# Dose-Dependent Effect of a New Biotin Compound in Hippocampal Remyelination in Rats

**DOI:** 10.1007/s12035-025-04686-y

**Published:** 2025-01-16

**Authors:** Burak Yulug, Ertugrul Kilic, Tuba Oğuz, Cemal Orhan, Besir Er, Mehmet Tuzcu, Ibrahim Hanifi Ozercan, Nurhan Sahin, Sinan Canpolat, James Komorowski, Sara Perez Ojalvo, Sarah Sylla, Seyda Cankaya, Kazim Sahin

**Affiliations:** 1 Department of Neurology, School of Medicine, Alaaddin Keykubat University, Alanya, Turkey; 2 Department of Physiology, Istanbul Medipol University, Istanbul, Turkey; 3 Research Institute for Health Sciences and Technologies (SABITA), Istanbul Medipol University, Istanbul, Turkey; 4 Department of Neuroscience, Graduate School of Health Sciences, Istanbul Medipol University, Istanbul, Turkey; 5 Department of Nutrition, Faculty of Veterinary Medicine, Firat University, Elazig, Turkey; 6 Department of Biology, Faculty of Science, Firat University, Elazig, Turkey; 7 Department of Pathology, Faculty of Medicine, Firat University, Elazig, Turkey; 8 Department of Physiology, Faculty of Medicine, Firat University, Elazig, Turkey; 9 Research and Development, JDS Therapeutics, LLC, Purchase, NY 10577 USA

**Keywords:** Biotin, Magnesium-biotin, Demyelination, Multiple sclerosis, Remyelination

## Abstract

Demyelination is commonly observed in neurodegenerative disorders, including multiple sclerosis (MS). Biotin supplementation is known to stabilize MS progression. To reduce the effective dose of biotin, we synthesized a new and superior form of biotin, a complex of magnesium ionically bound to biotin (MgB) and compared its dose-dependent effect with biotin alone after inducing demyelination using lysolecithin (LPC) in rats. Myelination was assessed using luxol fast blue staining and immunostaining against MBP protein, revealing that the most significant remyelination occurred in the MgB groups. Additionally, both biotin and MgB-treated animals showed dose-dependent improvements in spatial memory. Moreover, we detected a decrease in inflammatory proteins in both treatment groups, which was more prominent in high-dose MgB-treated animals and correlated with decreased expression of NF-κB p65, OP, and MMP-9 proteins. Further analysis of biotin-related proteins demonstrated that both biotin and, notably, MgB reversed the demyelination-dependent reduction of these proteins. Furthermore, biotin, particularly MgB, improved neuronal transmission proteins, Synapsin-1, PSD-93, and PSD-95. Additionally, both treatment groups exhibited increased BDNF, GAP43, and ICAM levels, with significant increments observed in high-dose MgB-treated animals. Increased GFAP, indicative of reactive gliosis, was observed in LPC-treated animals, and this effect was notably reversed by high-dose MgB treatment. The current data emphasize the dose-dependent beneficial effect on the remyelination process. Furthermore, the combination of biotin with Mg resulted in a more potent effect compared to biotin by itself. The strong influence of MgB encourages proof-of-concept studies using MgB in patients with MS.

**Supplementary information:**

The online version contains supplementary material available at 10.1007/s12035-025-04686-y.

## Introduction

Recent studies have highlighted the significant roles of oligodendrocyte damage, axonal degeneration, and demyelination in the pathophysiology of Multiple Sclerosis (MS) [1–3]. Clinical data from various sources have also demonstrated that the accumulation of axonal damage is closely linked to persisting demyelination and progressive neurological dysfunction in patients with the disease [1–5]. These findings collectively indicate that neurodegeneration is a substantial part of pathogenesis of MS, closely linked to prominent neuroinflammation, marked by the activation of reactive astrocytes and the migration of inflammatory cells. These processes encompass the upregulation of adhesion molecules and chemokines, along with an increase in matrix metalloproteinases (MMPs) like MMP-2, 7, 9, and 12 [4, 6, 7].

In experimental models of multiple sclerosis (MS), immune cells have been shown to influence the trafficking of T lymphocytes by triggering the activation of the receptor activator of NF-κB ligand (RANKL) [8, 9]. Interestingly, osteoprotegerin (OPG) and numerous other inflammatory proteins have also been shown to modulate the inflammatory response by interacting with NF-κB ligands in MS [9–11]. Furthermore, brain-derived neurotrophic factor (BDNF) enhances central nervous system (CNS) myelination during development and promotes remyelination and synaptogenesis following demyelination in animal models of MS [12, 13]. Its anti-inflammatory impact via NF-κB signaling pathways [14–17] further underscores its role. Consistent with these findings, increased levels of BDNF expression in humans with MS suggest a compensatory mechanism to promote the recovery/repair of damaged neurons [18–20].

Most clinical trials for MS that involve the use of established disease-modifying drugs have not primarily highlighted or documented significant cognitive improvements [21]. It remains challenging to explain why cognitive dysfunction responds poorly to pharmacological interventions. A possible reason might be that cognition, as a distinct function within the CNS, has extensive energy needs, and any disruption in the CNS might relate to varied metabolic dysfunctions both regionally and systemically. Reinforcing this idea, there's substantial evidence indicating that boosting bioenergetics in susceptible areas, like the hippocampus, could lead to marked enhancements in cognitive performance [22].

Biotin, given its recently discovered wide-ranging brain-protective effects in animal studies, along with its clinically acceptable therapeutic and safety profile, is emerging as a promising candidate for additional evaluation in the treatment of MS [23]. Several preclinical studies have shown that biotin exhibits significant remyelinating, neuroprotective, and energy-boosting activities [1]. Notably, daily oral biotin administration has shown to reduce the disability progression in MS patients, as observed in recent clinical trials involving 136 patients [24, 25]. These encouraging findings support the recent studies showing that biotin may additionally act as an essential promoter of carboxylases such as pyruvate carboxylase (PC), 3-methylcrotonyl-CoA carboxylase (MCC), propionyl-CoA carboxylase (PCC), and the two isoforms of acetyl-CoA carboxylase (ACC1 and ACC2), which increase aerobic energy production in neurons and oligodendrocytes [1, 2, 26].

While numerous experimental [27] and clinical studies have noted positive effects of biotin and magnesium individually on neuroinflammation and the associated allergic encephalomyelitis process [24, 25, 28, 29], and low levels of both substances have been detected in MS patients [30–32], there have been, to the best of our knowledge, no prior reports on a therapy that combines magnesium and biotin in an MS model. Moreover, there has been no prior research specifically assessing the potential cognitive-enhancing effects of biotin and magnesium in a model like this, particularly focusing on the hippocampus region, which is involved in MS-related cognitive dysfunction. Based on these unique mechanisms of action and previous inconsistent clinical observations [1, 23–25, 33], along with current therapeutic challenges in MS-related cognitive dysfunction, the current study was structured to evaluate whether a magnesium-biotin complex, or biotin alone, could reverse cognitive impairment in rats with hippocampal demyelination induced by LPC.

The magnesium biotinate (MgB) used in this study is a novel and unique complex, where magnesium is ionically bound to biotin. This complex is distinctive because it enhances biotin solubility by 40 times more than that of D-biotin [34]. Therefore, this study focused on the impacts of both high and low doses of magnesium biotinate (MgB) on remyelination, brain energy production, neuroinflammation, and cognitive-neurobehavioral assessments.

## Material and Methods

### Animals and Experimental Design

The study was conducted following National Institutes of Health (NIH) guidelines for the care and use of laboratory animals and was approved by the local government authorities (Firat University Animal Research Ethics Committee, Approved number: 257169). All rats were housed at a temperature of 22–24 °C with a constant 12-h light (07:00–19:00 h) and dark (19:00–07:00 h) cycle. A total of 42 male Wistar albino rats weighing 250–300 g were randomly divided into six groups (*n* = 7 per group). (i) Vehicle control group: 1.5 µl isotonic saline (LPC solvent) was injected into both hippocampi, and 5 days later, animals received 1 ml isotonic saline per oral gavage as a solvent of biotin for 4 weeks. (ii) LPC group: 1.5 µl of LPC 1% was injected into both hippocampi, and 5 days later, animals received 1 ml isotonic saline per oral gavage as a solvent of biotin/MgB for 4 weeks. (iii) Biotin1 (B1) group: 1.5 µl of LPC 1% was injected into both hippocampi, and 5 days later, animals received 0.9 mg/rat/day biotin in 1 ml isotonic saline per oral gavage for 4 weeks. (iv) Biotin2 (B2) group: 1.5 µl of LPC 1% was injected into both hippocampi, and 5 days later, animals received 9 mg/rat/day biotin in 1 ml isotonic saline per oral gavage for 4 weeks. (v) MgB1 group: 1.5 µl of LPC 1% was injected into both hippocampi, and five days later, animals received 0.9 mg/rat/day MgB in 1 ml isotonic saline per oral gavage for 4 weeks. (vi) MgB2 group: 1.5 µl of LPC 1% was injected into both hippocampi, and 5 days later, animals received 9 mg/rat/day MgB in 1 ml isotonic saline per oral gavage for 4 weeks. The amounts of biotin and MgB were calculated based on the 30 mg/day or 300 mg/day needed for a 70-kg adult human after adjusting doses based on metabolic body size [35, 36]. MgB was provided by JDS Therapeutics, LLC (Purchase, NY, USA) and synthesized as detailed previously (Patent no: AU2017318672A).

### Induction of Demyelination

As previously described, demyelination was induced in rat hippocampi by stereotaxic injection of lysolecithin (LPC) [37]. The animals were anesthetized and placed in a stereotaxic frame in a flat skull position. The skull was drilled, and a single dose of 1.5 µl LPC 1% (Sigma, St. Louis, MO, USA) in 0.9% saline was injected bilaterally into the CA1 area of hippocampi, using appropriate stereotaxic coordinates (AP = − 3.6 from bregma; ML = ± 1.6; DV = − 3.2 from dura surface) [37]. For the control rats, an equal volume of sterile saline was injected into the exact stereotaxic coordinates. The needle was held in place for an additional 3 min for the diffusion of LPC and to prevent the possible reflux of the solution through the needle tract.

### Analyses of Spatial Memory and Learning

For this purpose, the Morris water maze test was conducted as previously defined [38]. In brief, a circular black pool with a diameter of 160 cm and colored water (24 ± 2 °C) with a non-toxic black dye was used to contrast rats. A 12-cm-diameter black-colored round platform was submerged 1.5 cm below the water surface. Each rat underwent a daily session of four trials for six consecutive days. Before each trial, all rats were placed in the water maze room for 1 h. During the trial, rats were given 60 s to find the hidden platform and were allowed to remain on the platform for 30 s. If a rat failed to find the platform within 60 s, they were gently placed on the platform by the experimenter. The swimming pathway of the rats and the time spent finding the hidden platform were recorded for each trial.

At the end of the study, blood samples were collected by cardiac puncture from rats after an overnight fasting. Subsequently, all rats were sacrificed by cervical dislocation. Brains were immediately removed after the sacrification.

### Analyses of Biochemical Parameters

Serum glucose, total cholesterol, triglyceride, AST, ALT, creatinine, and urea levels were analyzed using a biochemical analyzer (Samsung Electronics Co, Suwon, Korea). To determine serum and brain magnesium levels, 0.3 g of the brain tissue and 0.5 ml of serum samples were digested with 5 ml concentrated nitric acid (65%, Merck, Darmstadt, Germany) in a Microwave Digestion System (Berghof, Eningen, Germany) for 30 min and then diluted 1∶10 with distilled deionized water (ddH_2_O). Lanthanum chloride (1%, Merck, Darmstadt, Germany) was added as an interference suppressant for Mg analyses. The heating program described in the oven’s user manual was employed. Mg levels were measured using atomic absorption spectrometry (AAS, Perkin‐ Elmer, Analyst 800, Norwalk, CT, USA) with flame atomization in an acetylene-air via recognized and fully confirmed procedures at the 285.2 nm wavelength with Zeeman background correction. The method was verified with certified reference materials (bovine muscle BCR 184), and the accuracy was 2% [39].

Samples were ultrafiltered for serum biotin level assay as previously described [40]. Tissue samples (300 mg) were processed in ice-cold homogenization buffers and then centrifuged. Before the biotin measurement, the supernatant was ultrafiltered. The pellet was dissolved in a homogenization buffer, and its concentration was adjusted to 40 g/l. The tissue samples were rapidly frozen in a mixture of dry ice and isopropanol and stored at − 80 °C until analyses. The biotin levels were determined by HPLC (Shimadzu, Kyoto, Japan) as previously described, with minor modifications [41, 42]. A C18-ODS-3 column was used as a reversed-phase column, and the biotin-containing chromatography fractions were dried under a stream of nitrogen before the assessment. For malondialdehyde (MDA) analyses, brain tissues were homogenized in ice-cold phosphate buffer solution for 5 min using an ultrasonic and a mechanical homogenizer and then centrifuged. The protein content in the supernatant was determined using nanodrop spectrophotometry (MaestroGen, Las Vegas, NV, USA). Then, an HPLC apparatus of Shimadzu UV–vis SPD-10 AVP detector, a CTO-10 AS VP column, and 30 mM KH2PO4 and methanol (82.5: 17.5, v/v, pH 3.6) at a flow rate of 1.2 ml/min were used (Shimadzu, Japan). Column waste was monitored at 250 nm.

### Histopathological Study

Luxol fast blue staining was employed to assess demyelination and remyelination based on earlier studies [37, 43]. Hematoxylin and eosin (H&E) staining was used to evaluate the pathological changes. Brain samples were fixed with 4% paraformaldehyde at 4 °C for 24 h. After dehydration with different alcohol gradients, the samples were cleared by incubation in xylene, embedded in paraffin, and then blocked. Serial sagittal sections of 6 µm thickness were obtained using a microtome. The sections were stained with hematoxylin and eosin or luxol fast blue stain using standard protocols. Histopathological changes were evaluated by an independent researcher according to standard criteria.

### Immunohistochemistry

Antibodies against myelin basic protein (MBP) and glial fibrillary acidic protein (GFAP) were used to assess demyelination/remyelination status and evaluate the astrocyte population, respectively. Sagittal brain sections of 6-µm thickness were deparaffinated for immunohistochemical staining. The sections underwent sequential immersion in xylene for 20 min, followed by 100%, 96%, 90%, and 70% alcohol for 10 min, ddH_2_O for 5 min, and finally in 1xPBS for 5 min. Antigen retrieval was performed using a 10 mM citrate buffer by intermittently boiling sections for 10 min in a microwave. Next, the sections were incubated with a blocking buffer containing BSA, NGS, and Triton X-100 in 1xPBS for 45 min at room temperature (RT) and then with primary antibodies overnight (O/N) (rat anti-MBP 1/100, Bio-Rad; rabbit anti-GFAP 1/400, Thermo Fisher). Following washing steps with 1xPBS with Tween-20, the sections were incubated with secondary antibodies for 2 h at RT (goat anti-rat 555, 1/500, Jackson Immunoresearch; goat anti-rabbit 555, 1/500, AAT Bioquest iFlour). Antibodies were removed by washing steps with 1xPBS with Tween-20 and the sections were then mounted using Sigma Fluoromount Mounting Medium for imaging. Images from the CA1 region of the hippocampus were acquired by Zeiss LSM800 laser scanning confocal microscope as z-stacks using 20 × objective and 40 × objective with oil immersion.

### Western Blot Analysis

Proteins and their levels in the hippocampus were analyzed by Western blot [44]. Briefly, hippocampal tissue samples from each group were pooled and lysed in a buffer containing enzyme inhibitors, including phenylmethylsulfonyl fluoride, soybean trypsin protease inhibitor cocktail, and sodium fluoride as a phosphatase inhibitor. Triton X-100 and Tween-20 were used for cell lysate preparation to solubilize proteins and disrupt cell membranes. The total protein content was assessed using the Qubit 4.0 Fluorometer (Invitrogen, CA, USA). The target proteins in this study were suitable for a 12% resolving SDS-PAGE gel percentage for the Western Blot. 20-µg samples were electrophoresed and then transferred to a nitrocellulose membrane using the Trans-Blot Turbo Transfer System. Subsequently, the membranes were blocked for 1 h at RT, washed, and incubated O/N with the following primary antibodies: AC, ACC1, ACC2, PC, PCC, presynaptic Synapsin-I, postsynaptic PSD-95, PSD-93, IL-17A, IL-6, TNF-α, GFAP, GAP43, ICAM-1, BDNF, and CXCL-16 (Abcam, Cambridge, UK). Primary antibody dilutions varied, as indicated in the datasheets for each protein, with concentrations mostly ranging between 1/1000–1/3000 dilutions in a blocking solution (BSA). Afterwards, the membranes were washed with TBS-T and incubated with a rabbit secondary antibody (Abcam, Cambridge, UK) for 1 h at RT. Individual blots were performed at least three times. Protein loads were controlled by stripping and reprobing the nitrocellulose membrane with an anti-β-actin antibody (Abcam, Cambridge, UK). Protein levels were analyzed densitometrically using an image analysis system (ImageJ; National Institute of Health, Bethesda, MD, USA), corrected with values determined from β-actin blots, and expressed as relative values compared with the control group.

### Statistical Analysis

Statistical analysis was conducted using the SPSS software (version 15, SPSS Inc., Chicago, USA). The normality of data was assessed using the Shapiro–Wilk test. Then, a one-way analysis of variance (ANOVA) was performed to determine if there were any significant differences among the groups. To identify specific group differences, the Tukey post hoc test was applied. The results are presented as mean ± standard error of the mean (SEM) or standard deviation (SD) to provide a measure of the central tendency and variability of the data. Throughout the study, *p*-values less than 0.05 were considered statistically significant.

## Results

### Bodyweight and Biochemical Parameters

As shown in Table 1, the demyelination of the hippocampus resulted in a decrease in body weight when compared to the vehicle control. However, administering high doses of biotin, particularly both of MgB, alleviated weight loss in rats. There were no noticeable differences in levels of glucose, total cholesterol, triglycerides, AST, ALT, creatinine, and urea, as detailed in Table 1.

**Table 1:** Effects of biotin and magnesium biotinate (MgB) supplementation on body weight and various biochemical parameters in hippocampal demyelinated rats

Items	Groups					
	**Control**	**LPC**	**LPC + B1**	**LPC + B2**	**LPC + MgB1**	**LPC + MgB2**
Body weight, g	290.71 ± 5.55	251.33 ± 7.26****	259.57 ± 2.81***	264.67 ± 1.38**	272.57 ± 1.99*^,#^	281.86 ± 3.02^###,&&^
Glucose, mg/dL	89.71 ± 3.46	87.73 ± 3.65	87.41 ± 5.08	91.45 ± 3.79	89.43 ± 4.00	86.71 ± 3.40
T-C, mg/dl	76.61 ± 4.83	76.30 ± 4.45	76.16 ± 5.17	74.78 ± 4.20	70.94 ± 4.30	71.50 ± 1.96
Triglyceride, mg/dl	57.71 ± 4.62	58.17 ± 4.81	56.71 ± 3.15	57.83 ± 2.02	57.29 ± 3.89	55.57 ± 1.48
AST, U/L	230.29 ± 8.58	241.67 ± 13.15	238.86 ± 11.16	236.83 ± 8.83	233.86 ± 11.16	232.00 ± 9.87
ALT, U/L	60.29 ± 4.42	66.83 ± 6.82	67.57 ± 7.12	64.83 ± 5.79	62.71 ± 4.13	63.29 ± 4.37
Creatinine, mg/dl	0.25 ± 0.01	0.27 ± 0.02	0.26 ± 0.01	0.26 ± 0.01	0.24 ± 0.01	0.24 ± 0.02
Urea, mg/dl	26.01 ± 5.11	28.85 ± 4.59	28.56 ± 4.26	25.10 ± 3.80	25.86 ± 2.51	24.86 ± 1.64

*LPC* lysolecithin, *B1* Biotin 0.9 mg/rat/day, *B2* Biotin 9 mg/rat/day, *MgB1* magnesium biotinate (Biotin 0.9 mg/rat/day), *MgB2* magnesium biotinate (Biotin 9 mg/rat/day), *T-C* total cholesterol, *ALT* Alanine aminotransferase, *AST*, Aspartate aminotransferase. Data presented as mean and standard error. Statistical significance between groups were shown as compared to Control *p < 0.05, **p < 0.01, ***p < 0.001, ****p < 0.0001; compared to LPC ^#^*p* < 0.05, ^###^ *p* < 0.001; compared to LPC + B1; ^&&^*p* < 0.01, using ANOVA and Tukey’s *post-hoc* test

In demyelinated rats (LPC group), both serum and brain tissue demonstrated lower levels of Mg when compared to the control rats, as shown in Table 2. Interestingly, not only were Mg levels reduced, but biotin levels in both serum and brain tissue also decreased in the demyelinated rats. This decline was rectified by supplementing with both biotin and MgB Furthermore, we examined the levels of malondialdehyde (MDA), discovering an increase in the LPC group of demyelinated rats. However, this rise in MDA levels was successfully counteracted by treatments with biotin and especially MgB (Table 2).

**Table 2:** Effects of biotin and magnesium biotinate (MgB) supplementation on serum and brain magnesium, biotin and malondialdehyde concentrations in hippocampal demyelinated rats

Items	Groups					
	**Control**	**LPC**	**LPC ± B1**	**LPC ± B2**	**LPC ± MgB1**	**LPC ± MgB2**
Serum Mg, mg/dL	2.85 ± 0.07	2.11 ± 0.03****	2.17 ± 0.04****	2.09 ± 0.04****	3.62 ± 0.04****^,####,&&&&,$$$$^	6.01 ± 0.04****^,####,&&&&,$$$$,++++^
Brain Mg, µg/g	150.70 ± 2.44	115.57 ± 3.96****	119.41 ± 1.71****	118.38 ± 3.58****	161.58 ± 0.57^####,&&&&,$$$$^	187.77 ± 2.33****^,####,&&&&,$$$$,++++^
Serum biotin, nmol/L	62.53 ± 2.62	34.47 ± 1.25****	57.56 ± 1.92^####^	81.92 ± 2.15****^,####,&&&&^	65.13 ± 1.93^####,$$$$^	102.17 ± 2.82****^,####,&&&&,$$$$,++++^
Brain biotin, nmol/g	0.182 ± 0.002	0.104 ± 0.005****	0.151 ± 0.001****^,####^	0.187 ± 0.003^####,&&&&^	0.168 ± 0.001**^,####,&&,$$$^	0.199 ± 0.002**^,####,&&&&,$,++++^
Serum MDA, nmol/mL	1.30 ± 0.04	3.08 ± 0.11****	2.70 ± 0.05****^,##^	2.53 ± 0.07****^,####^	2.21 ± 0.04****^,####,&&&&,$^	1.84 ± 0.04****^,####,&&&&,$$$$,++^
Brain MDA, nmol/g	2.92 ± 0.05	7.37 ± 0.21****	6.25 ± 0.08****^,####^	5.84 ± 0.07****^,####^	5.10 ± 0.10****^,####,&&&&,$$$^	4.55 ± 0.09****^,####,&&&&,$$$$,+^

*LPC* lysolecithin, *B1* Biotin 0.9 mg/rat/day, *B2* Biotin 9 mg/rat/day, *MgB1* magnesium biotinate (Biotin 0.9 mg/rat/day), *MgB2* magnesium biotinate (Biotin 9 mg/rat/day); *MDA* Malondialdehyde. Data presented as mean and standard error. Statistical significance between groups were shown as compared to Control **p < 0.01, ****p < 0.0001; compared to LPC ^##^*p* < 0.01, ^####^*p* < 0.0001; compared to LPC + B1; ^&&^*p* < 0.01, ^&&&&^*p* < 0.0001; compared to LPC + B2; ^$^*p* < 0.01, ^$$$^*p* < 0.001, ^$$$$^*p* < 0.0001; compared to LPC + MgB1 ^+^*p* < 0.05, ^++^*p* < 0.01, ^++++^*p* < 0.0001, using ANOVA and Tukey’s *post-hoc* test

### Spatial Memory and Learning

#### Entries to Target Quadrant and Probe Trial in the Morris Water Maze

**Fig. 1:**
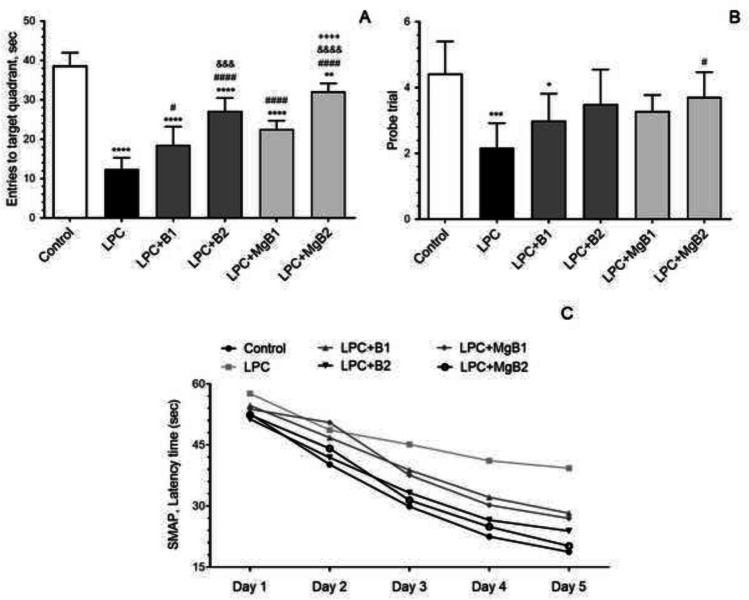
Effects of biotin and magnesium biotinate (MgB) supplementation on entries to target quadrant (**A**), probe trial (**B**), and latency (**C**) to find the hidden platform from the first day to the fifth day in the Morris water maze task in hippocampal demyelinated rats (*n* = 7). Different symbols (*, #, &, $, and +) indicate difference compared to the Control, LPC, LPC + B1, LPC + B2, LPC + MgB1, and LPC + MgB2 groups, respectively) Statistical significance between groups was shown as compared to Control *p < 0.05, **p < 0.01, ***p < 0.001, ****p < 0.0001; compared to LPC ^#^*p* < 0.05, ^####^*p* < 0.0001; compared to LPC + B1; ^&&&^*p* < 0.001, ^&&&&^*p* < 0.0001; compared to LPC + MgB1; ^++++^*p* < 0.0001, respectively, using ANOVA and Tukey’s *post-hoc* test. B1: Biotin 0.9 mg/rat/day; B2: Biotin 9 mg/rat/day; MgB1: magnesium biotinate (Biotin 0.9 mg/rat/day); MgB2: magnesium biotinate (Biotin 9 mg/rat/day)

We evaluated the effects of supplementing biotin and MgB on the performance in the target quadrant and probe trial of the Morris water maze task. The data showed that impaired behavioral parameters resulting from demyelination were significantly and dose-dependently improved after the administration of biotin and MgB (Fig. 1A, B; LPC + B1 *vs.* LPC + B2, *p* < 0.05; LPC + MgB1 *vs.* LPC + MgB2, *p* < 0.05).

Latency to Find the Hidden Platform in the Morris Water Maze Task.

We examined the impact of biotin and MgB supplementation on the on the latency to find the concealed platform in the Morris water maze task, spanning from the first to the fifth day. The findings indicated that the behavioral shortcomings caused by demyelination were substantially improved in a manner that was both significant and dependent on the dosage, following the treatment with biotin and MgB (Fig. 1C; LPC + B1 vs. LPC + B2, *p* < 0.05; LPC + MgB1 vs. LPC + MgB2, *p* < 0.05).

### Histopathology

**Fig. 2:**
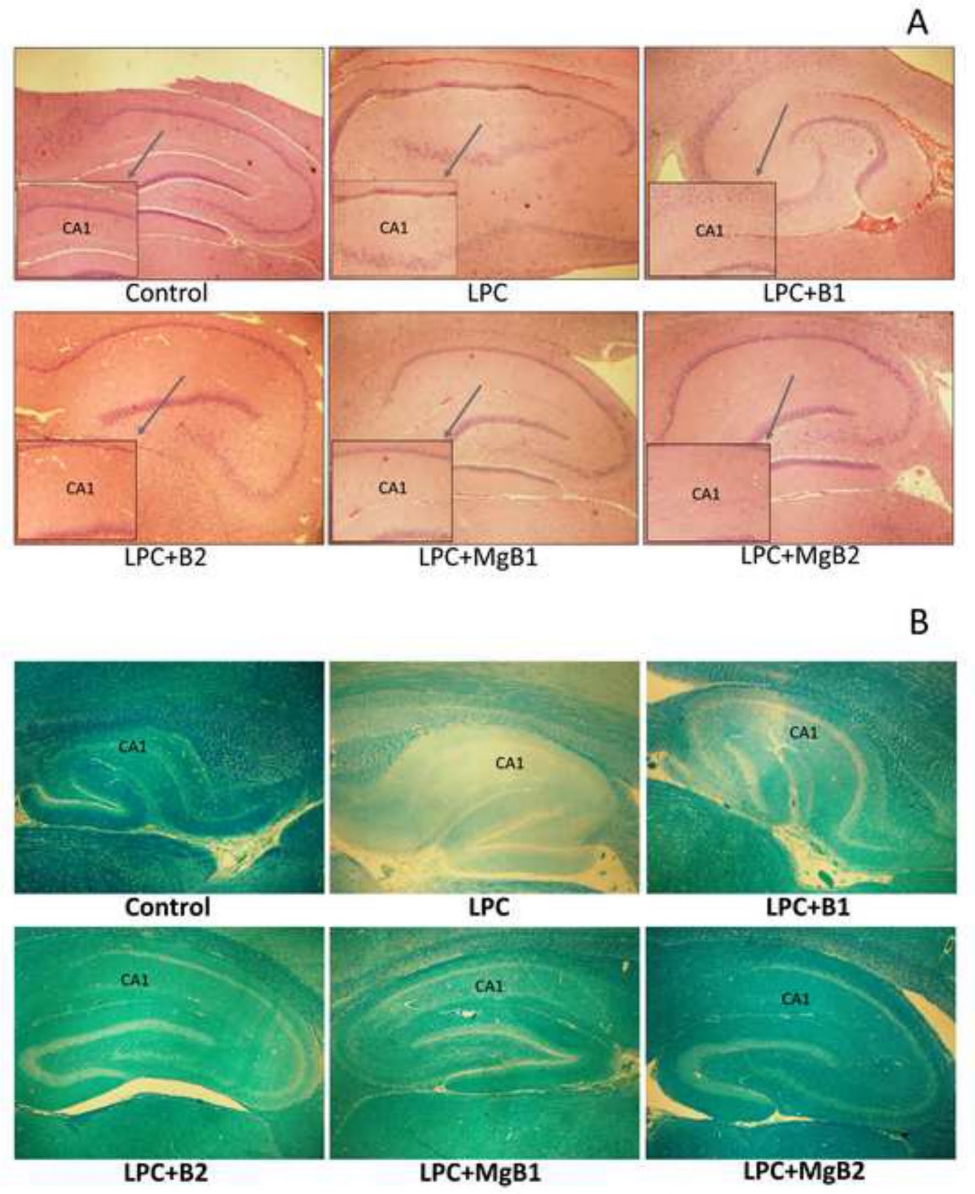
Representative images of histopathological changes stained by H&E (**A**) and Luxol fast blue (**B**) in response to Biotin B1, B2, MgB1, and MgB2 treatments in demyelinated rat hippocampus. (*n* = 7). B1: Biotin 0.9 mg/rat/day; B2: Biotin 9 mg/rat/day; MgB1: magnesium biotinate (Biotin 0.9 mg/rat/day); MgB2: magnesium biotinate (Biotin 9 mg/rat/day)

Initially, we observed that LPC administration resulted in diffuse hippocampal degeneration, as evidenced by H&E staining (Fig. 2A). In the LPC group, there was slight edema, infrequent lymphocytic infiltration, and remarkable degeneration of pyramidal cells around the CA1 region of the hippocampus. Conversely, in the LPC + B1 and LPC + B2 groups, we detected mild edema and mild degeneration of pyramidal cells around the CA1 region. However, the LPC + MB1 group showed only mild edema. Notably, the MgB2 group exhibited a normal histological appearance of the hippocampus.

In saline-treated controls, no hippocampal demyelination was observed, whereas LPC induced significant demyelination, primarily around the CA1 regions in LPC-treated animals, compared to the control group (Fig. 2B). Biotin and MgB treatment at both doses accelerated remyelination in LPC treated animals. Furthermore, the MgB2 group exhibited complete remyelination (Fig. 2B).

### Immunohistochemistry

**Fig. 3:**
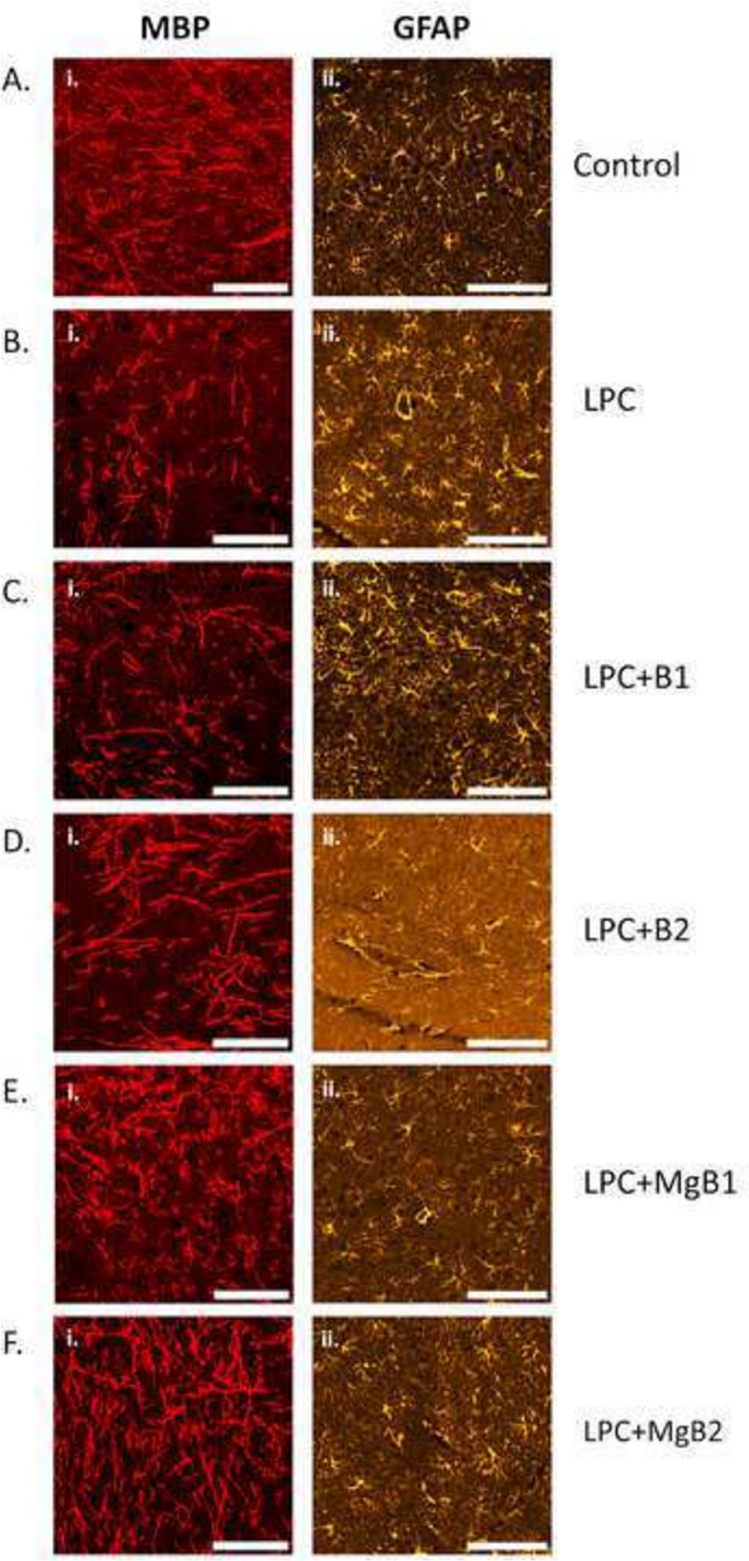
Representative images of gliosis in response to Biotin B1, B2, MgB1, and MgB2 treatments in demyelinated rat hippocampus. The left panel shows immunohistochemical staining against myelin basic protein (MBP), labeling myelin (red), and the right panel shows immunohistochemical staining against the glial fibrillary basic protein (GFAP), labeling astrocytes (orange). **B.i** LPC causes extensive demyelination in the rat hippocampus, compared to the saline-treated group in (**A.i**). Both doses of biotin accelerate remyelination; however, complete remyelination is not observed (**C.i** and **D.i**). An increase in the astrocyte population is observed in the LPC-treated group (**B.ii**) compared to saline-treated control group (**A.ii**). The reactive state of astrocytes continues at a low dose of biotin (**C.ii**) but decreases when a high dose is applied (**D.ii**). MgB1 and MgB2 also accelerate remyelination (**E.i** and **F.i**) and attenuate reactive states (**E.ii** and **F.ii**). Almost complete remyelination is observed in these groups (**E** and **F**). Images were taken from the CA1 region of the hippocampus with 40 × (i) and 20 × (ii) objectives. Scale bars: i, 50 µm; ii, 100 µm

LPC causes extensive demyelination in the rat hippocampus, which remains observable even after 4.5 weeks of LPC injection (Fig. 3B.i). Additionally, we observed an increased population of astroglial cells in the demyelination area, indicating reactive gliosis (Fig. 3B.ii) compared to the saline-administered control (Fig. 3A.ii). Both doses of biotin cause accelerated remyelination, although it is partial (Fig. 3C.i and D.i) compared to the control (Fig. 3B.i). The low dose of biotin did not alter the GFAP + astrocyte population, suggesting the continuity of the reactive state (Fig. 3C.ii). However, the high dose of biotin decreased the GFAP + astrocyte population (Fig. 3D.ii). Furthermore, both MgB1 and MgB2 treatments appear to be more successful in gliosis; they accelerate remyelination and attenuate reactive gliosis (Fig. 3E and F) compared to biotin treatments (Fig. 3C and D). Figure 3A.i received only saline injection in the hippocampi; and demyelination was not observed. Interestingly, both MgB1 (Fig. 3E.i) and MgB2 (Fig. 3F.i) treatments for 4 weeks restored myelin levels similar to the saline-treated control group in Fig. 3A.i. However, at the same time point, demyelination was still observable in Fig. 3B.i.

### Inflammatory Markers

**Fig. 4:**
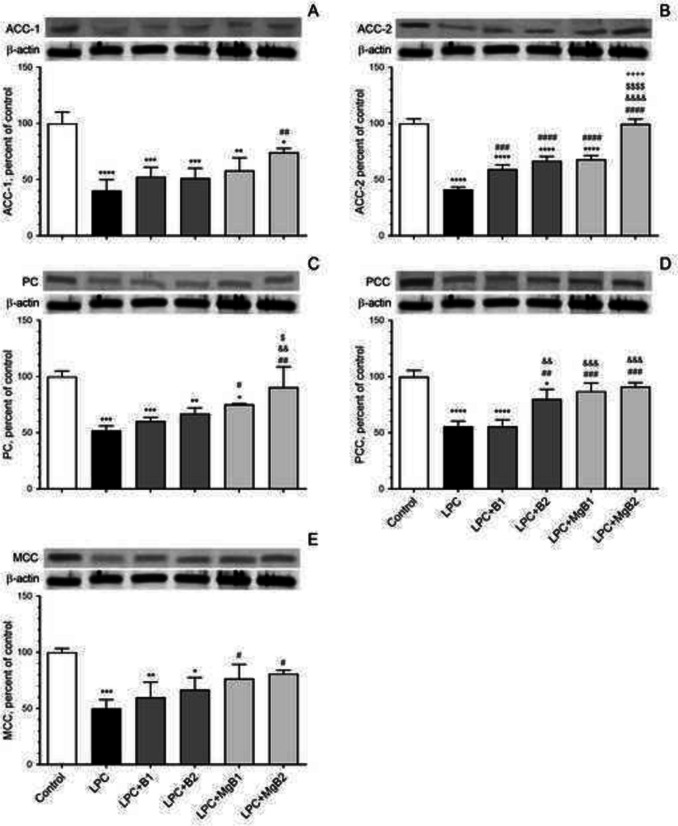
Effects of biotin and magnesium biotinate (MgB) supplementation on hippocampal interleukin 6 (IL-6, **A**), interleukin 17A (IL-17A, **B**), tumor necrosis factor-alpha (TNF-α, **C**), nuclear factor kappa light chain enhancer of activated B cells (NF-κB p65, **D**), chemokine (C–C motif) ligand 3 (CCL-3, **E**), chemokine (C–C motif) ligand 5 (CCL-5, **F**) and chemokine (C-X-C motif) ligand 16 (CXCL-16, **G**), osteoprotegerin (OPG, **H**) and matrix metallopeptidase 9 (MMP-9, **I**) protein levels in hippocampal demyelinated rats (*n* = 7). Data are expressed as a percent of the control value. Each bar represents the mean and standard error of the mean. Blots were repeated at least three times. Western blot analysis was performed with actin included, ensuring equal protein loading. The data are percentages of the control. Different symbols (*, #, &, $, and + indicates difference compared to the Control, LPC, LPC + B1, LPC + B2, LPC + MgB1, and LPC + MgB2 groups, respectively). Statistical significance between groups was shown as compared to Control *p < 0.05, **p < 0.01, ***p < 0.001, ****p < 0.0001; compared to LPC ^#^*p* < 0.05, ^##^*p* < 0.01, ^###^*p* < 0.001, ^####^*p* < 0.0001; compared to LPC + B1; ^&^*p* < 0.05, ^&&^*p* < 0.01, ^&&&^*p* < 0.001, ^&&&&^*p* < 0.0001; compared to LPC + B2; ^$^*p* < 0.05, ^$$^*p* < 0.01, ^$$$^*p* < 0.001, ^$$$$^*p* < 0.0001; compared to LPC + MgB1 ^+^*p* < 0.05, ^+++^*p* < 0.001, ^++++^*p* < 0.0001, using ANOVA and Tukey’s *post-hoc* test. B1, Biotin 0.9 mg/rat/day; B2, Biotin 9 mg/rat/day; MgB1, magnesium biotinate (Biotin 0.9 mg/rat/day); MgB2, magnesium biotinate (Biotin 9 mg/rat/day)

We examined the effects of biotin and MgB supplementation on inflammatory parameters in the hippocampus. Our findings revealed that levels of IL-6, IL-17A, TNF-α, chemokine (C–C motif) ligand 3 (CCL-3), chemokine (C–C motif) ligand 5 (CCL-5), and chemokine (C-X-C motif) ligand 16 (CXCL-16) were significantly and dose-dependently reduced following the application of B1, B2, MgB1, and MgB2 (Fig. 4A, B, C, E, F, G). However, IL-17A and CCL-5 levels showed no statistically significant differences between the LPC + B1 and LPC + B2 groups, and LPC + MgB1 and LPC + MgB2 groups, respectively (Fig. 4B, F; *p* > 0.05).

Further analysis on NF-κB, osteoprotegerin (OPG), and matrix metallopeptidase 9 (MMP-9) revealed that NF-κB p65 levels were significantly and dose-dependently decreased within the groups (Fig. 4D; LPC + B1 *vs*. LPC + B2 and LPC + MgB1 *vs*. LPC + MgB2, *p* < 0.05). In contrast, both OPG (Fig. 4H) and MMP-9 (Fig. 4I) levels were unaffected by increasing biotin dosage (LPC + B1 *vs.* LPC + B2, *p* > 0.05).

Notably, OPG levels responded well to the combination therapy and showed statistically increased levels after increasing the biotin dosage in the MgB2 group (Fig. 4H; LPC + MgB1 *vs.* LPC + MgB2, *p* < 0.05).

### Biotin-Related Enzymes

**Fig. 5:**
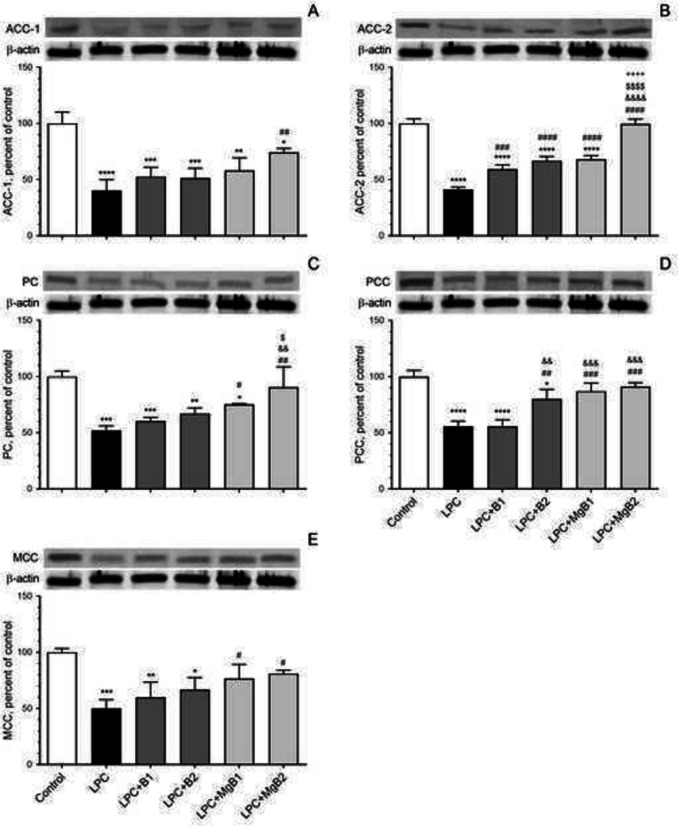
Effects of biotin and magnesium biotinate (MgB) supplementation on hippocampal acetyl-CoA carboxylase 1 (ACC1, **A**), acetyl-CoA carboxylase 2 (ACC2, **B**), pyruvate carboxylase (PC, **C**), propionyl-CoA carboxylase (PCC, **D**), and 3-methylcrotonyl-CoA carboxylase (MCC, **E**) protein levels in hippocampal demyelinated rats (*n* = 7). Data are expressed as a percent of the control value. Each bar represents the mean and standard error of the mean. Blots were repeated at least three times. Western blot analysis was performed with actin included, ensuring equal protein loading. The data are percentages of the control. Different symbols (*, #, &, $, and +) indicates difference compared to the Control, LPC, LPC + B1, LPC + B2, LPC + MgB1, and LPC + MgB2 groups, respectively). Statistical significance between groups were shown by as compared to Control *p < 0.05, **p < 0.01, ***p < 0.001, ****p < 0.0001; compared to LPC ^#^*p* < 0.05, ^##^*p* < 0.01, ^###^*p* < 0.001, ^####^*p* < 0.0001; compared to LPC + B1; ^&&^*p* < 0.01, ^&&&^*p* < 0.001, ^&&&&^*p* < 0.0001; compared to LPC + B2; ^$^*p* < 0.05, ^$$$$^*p* < 0.0001; compared to LPC + MgB1.^++++^*p* < 0.0001, using ANOVA and Tukey’s *post-hoc* test. B1, Biotin 0.9 mg/rat/day; B2, Biotin 9 mg/rat/day; MgB1, magnesium biotinate (Biotin 0.9 mg/rat/day); MgB2, magnesium biotinate (Biotin 9 mg/rat/day)

We assessed the effects of biotin and MgB supplementation on acetyl-CoA carboxylase 1 (ACC1), acetyl-CoA carboxylase 2 (ACC2), pyruvate carboxylase (PC), propionyl-CoA carboxylase (PCC), 3-methylcrotonyl-CoA carboxylase (MCC). No dose-dependent increases were observed in biotin-related enzymes following the application of low (B1) and high doses of biotin (B2) (Fig. 5A–E; LPC + B1 *vs*. LPC + B2, *p* > 0.05) except the PCC group which showed no statistically significant increase after increasing the biotin dosage (Fig. 5D; LPC + B1 *vs*. LPC + B2, *p* < 0.05).

Comparing the effects of MgB1 and MgB2 on biotin-related enzymes, we observed significant increases in all biotin-related enzymes (Fig. 5A–E, p < 0.05) except for the PCC (Fig. 5D, LPC + B1 *vs*. LPC + B2, *p* > 0.05) and MCC enzymes (Fig. 5E; LPC + B1 *vs*. LPC + B2, *p* > 0.05) in the only biotin-administered groups.

### BDNF, GAP43, GFAP, and ICAM Proteins

**Fig. 6:**
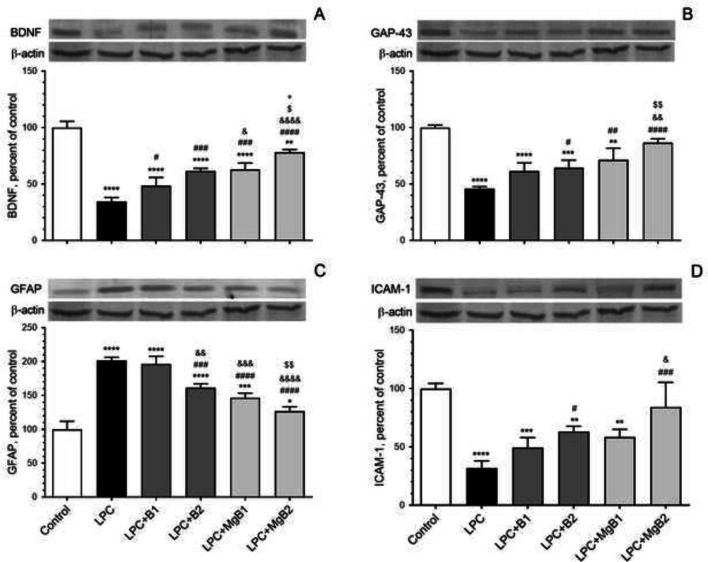
Effects of biotin and magnesium biotinate (MgB) supplementation on hippocampal brain-derived neurotrophic factor (BDNF, **A**), growth-associated protein (GAP43, **B**), glial fibrillary acidic protein (GFAP, **C**), and intercellular adhesion molecule 1 (ICAM-1, **D**) protein levels in hippocampal demyelinated rats (*n* = 7). Data are expressed as a percent of the control value. Each bar represents the mean and standard error of the mean. Blots were repeated at least three times. Western blot analysis was performed with actin included, ensuring equal protein loading. The data are percentages of the control. Different symbols (*, #, &, $, and +) indicates difference compared to the Control, LPC, LPC + B1, LPC + B2, LPC + MgB1, and LPC + MgB2 groups, respectively). Statistical significance between groups were shown as compared to Control *p < 0.05, **p < 0.01, ***p < 0.001, ****p < 0.0001; compared to LPC ^#^*p* < 0.05, ^##^*p* < 0.01, ^###^*p* < 0.001, ^####^*p* < 0.0001; compared to LPC + B1; ^&^*p* < 0.05, ^&&^*p* < 0.01, ^&&&^*p* < 0.001, ^&&&&^*p* < 0.0001; compared to LPC + B2; ^$^*p* < 0.05, ^$$^*p* < 0.01; compared to LPC + MgB1.^+^*p* < 0.05, using ANOVA and Tukey’s *post-hoc* test. B1, Biotin 0.9 mg/rat/day; B2, Biotin 9 mg/rat/day; MgB1, magnesium biotinate (Biotin 0.9 mg/rat/day); MgB2, magnesium biotinate (Biotin 9 mg/rat/day)

We evaluated the impact of biotin and MgB supplementation on BDNF, GAP43, GFAP, and ICAM, finding that both biotin and MgB supplementation led to a dose-dependent increase in the levels of the growth factor BDNF and GAP43 (Fig. 6A, B; LPC + B1 *vs*. LPC + B2, *p* < 0.05) and MgB supplementation (Fig. 6A, B; LPC + MgB1 *vs*. LPC + MgB2, *p* < 0.05). In contrast, GFAP levels were dose-dependently decreased in biotin-treated groups (Fig. 6C; LPC + B1 *vs*. LPC + B2, *p* < 0.05) and particularly in the MgB-treated groups (Fig. 6C; LPC + MgB1 *vs*. LPC + MgB2, *p* < 0.05).

### Synaptic Transmission Proteins

**Fig. 7:**
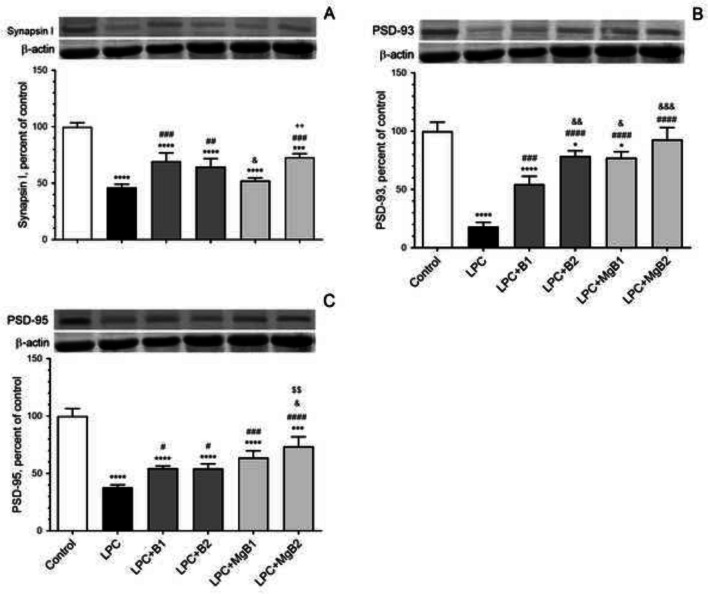
Effects of biotin and magnesium biotinate (MgB) supplementation on hippocampal Synapsin-I (**A**), postsynaptic density protein 93 (PSD-93, **B**) and postsynaptic density protein 95 (PSD-95, **C**) protein levels in hippocampal demyelinated rats (*n* = 7). Data are expressed as a percent of the control value. Each bar represents the mean and standard error of the mean. Blots were repeated at least three times. Western blot analysis was performed with actin included, ensuring equal protein loading. The data are percentages of the control. Different symbols (*, #, &, $, and +) indicates difference compared to the Control, LPC, LPC + B1, LPC + B2, LPC + MgB1, and LPC + MgB2 groups, respectively). Statistical significance between groups were shown as compared to Control *p < 0.05, ***p < 0.001, ****p < 0.0001; compared to LPC ^#^*p* < 0.05, ^##^*p* < 0.01, ^###^*p* < 0.001, ^####^*p* < 0.0001; compared to LPC + B1; ^&^*p* < 0.05, ^&&^*p* < 0.01, ^&&&^*p* < 0.001; compared to LPC + B2; ^$$^*p* < 0.01; compared to LPC + MgB1.^++^*p* < 0.01, using ANOVA and Tukey’s *post-hoc* test. B1, Biotin 0.9 mg/rat/day; B2, Biotin 9 mg/rat/day; MgB1, magnesium biotinate (Biotin 0.9 mg/rat/day); MgB2, magnesium biotinate (Biotin 9 mg/rat/day)

We assessed the effects of biotin and MgB supplementation on Synapsin-I (Fig. 7A), postsynaptic density protein 93 (PSD-93, Fig. 7B), and postsynaptic density protein 95 (PSD-95, Fig. 7C) in the hippocampus. Our results revealed that Synapsin-I was significantly increased in the LPC + MgB1 group compared to the LPC + MgB2 group (Fig. 7A). Moreover, the level of PSD-93 was considerably higher in the LPC + B2 group compared to the LPC + B1 group (Fig. 7B; *p* < 0.05). However, no significant change was observed in the PSD-95 level in the LPC + B2 group compared to the LPC + B1 group (Fig. 7C; *p* > 0.05). Notably, in the LPC + MgB2 group, the levels of all synaptic proteins were significantly increased compared to the LPC + MgB1 group (*p* < 0.05; Fig. 7).

## Discussion

In the present study, MgB significantly reduces inflammatory markers and oxidative stress while promoting the expression of synaptic transmission and axonal regeneration proteins and restoring critical biotin-related energetic enzymes, which correlates with improved cognitive scores. These results are consistent with previous studies highlighting the role of inflammation and oxidative stress in experimental autoimmune encephalitis (EAE). As an example, Alhazzini et al. [45] demonstrated that both innate and adaptive immune cells are involved in the pathology of MS, with the signal transducer and activator of transcription 3 (STAT3) signaling possibly playing a key part in the function of neutrophils and CD4 + T cells. Their research indicated that the suppression of STAT3 signaling led to an improvement in symptoms within the EAE model.

Similarly, the elevation in axonal regeneration proteins and the restoration of neurological and cognitive scores are consistent with findings from other EAE models. In an intriguing study, Ansari et al. revealed that cathepsin B inhibition reduced the Th17 and Th22 cell populations, suppressed Th1-related transcription factors, and improved clinical scores in mice. Additionally, they found that the inhibition of cathepsin signaling led to an increase in the expression of the neurofilament NF-H protein within brain tissue. This finding suggests a role for cathepsin B in the development and progression of MS, particularly through causing neuroaxonal damage [46]. Furthermore, our findings of improved neurobehavioral scores associated with decreased inflammatory parameters corroborate previous research on the role of inflammatory cytokines, such as interleukin IL-17A, which contributes to a depression-like state via the NF-κB and p38/MAPK signaling pathways in mice [47]. This situation has been shown to be alleviated with appropriate anti-inflammatory therapies [48, 49]. The observed correlation between improved neurobehavioral scores and decreased inflammatory parameters in our study aligns closely with existing literature on the role of inflammatory cytokines in neurobehavioral outcomes. This consistency with previous data further strengthens the validity and significance of our findings.

While several therapeutic options offer some short- and long-term immune-modulating effects, they often fail to provide clinically sustainable neuroprotective activities, and ultimately falling short in improving the long-term disease course in MS [24]. The challenge with translational MS studies lies in connecting preclinical results with complex human biological processes, involving interactions among multiple key pathophysiological components [50, 51]. The resulting pathophysiology is so intricate that therapeutic interventions targeting singular pathways may not yield sufficient benefits. This, in turn, led researchers to hypothesize that alternative medications, like nutritional supplements [52] which target multiple pathways, might be necessary to improve the disease course. Our present data demonstrate that a combination of magnesium and high-dose biotin is highly effective in accelerating remyelination, improving cognitive scores, and reducing inflammatory parameters (ILs, TNF-α, chemokines, NFκB, and GFAP) without causing any adverse effects in case of hippocampal demyelination. Notably, the magnesium and high-dose biotin groups showed enhanced cognitive scores, which were associated with considerably elevated bioenergetic mechanisms, including upregulation of important biotin-related enzymes implicated in cognitive processes, such as ACC1, ACC2, PC, PCC, and MCC.

It is worth noting that cognitive impairment (CI) is a commonly reported phenomenon in individuals diagnosed with multiple sclerosis (MS), with a prevalence rate ranging from 34 to 65% [53]. To date, the pathological brain changes associated with cognitive disability in MS are not fully understood, and despite the efficacy of disease-modifying treatments in preventing cognitive decline, the results of clinical trials have been disappointing [54, 55]. As mentioned earlier, a potential explanation for this treatment failure could be attributed to the physiological organization of cognitive networks within the brain. In this context, an increasing body of research suggests that brain networks associated with cognitive processes heavily rely on high levels of energy and demonstrate favorable cognitive responses to metabolic interventions [56–58]. This reinforces the therapeutic significance of our study, proposing magnesium and biotin as not only a multimodal energetic therapy option but also an effective pro-cognitive intervention in the context of neuroinflammatory degenerative diseases like MS.

Considering the previously demonstrated effects of BDNF on synaptogenesis, cognition, and learning, we also analyzed the expression of BDNF, GAP43, ICAM, and synaptic proteins (i.e., PSD-93, PSD-95, and Synapsin-I). Consistent with earlier studies [59–61], we observed that BDNF levels and markers for axonal regeneration and synaptic remodeling increased in a dose-dependent manner after treatment with biotin and MgB. We then explored whether the pro-cognitive potential of BDNF and other neuro-regeneration markers might still be sufficient to alter neurobehavioral outcomes in the whole experimental group. Not surprisingly, we observed that the biotin alone and MgB complex improved cognitive scores in a dose-dependent manner, consistent with the effects of BDNF on learning, memory, and depression [60, 62]. This might support the presence of interactive processes between remyelination, synaptogenesis, and (region-specific) energy requirements involved in improved neurological status. For instance, the different structural expression patterns of biotin-related enzymes and the varying energy requirements of neuronal structures may contribute to additive beneficial effects. A good example may be acetyl-CoA carboxylase 1 and acetyl-CoA carboxylase 2, which are expressed in oligodendrocytes and induce myelin repair by supporting fatty acid synthesis. On the other hand, other biotin-related enzymes are primarily expressed in neurons and protect axons by enhancing energy production 1, [63, 64].

Since demyelination starts shortly after the injection of LPC, it serves as a valuable agent for studying the effects of chemicals on demyelination and remyelination status. In rats, remyelination typically begins after 5 weeks of LPC treatment and is completed after 3 months [65, 66]. Therefore, this model is suitable for studying the effects of biotin and MgB doses on remyelination. To evaluate their effects, we administered the products starting from 5 dpi of LPC, where it is known that extensive demyelination is already observed in 3 dpi [65]. In our results, demyelination was still observable at 33 dpi (Fig. 3B.i), compared to the control group (Fig. 3A.i). Biotin and MgB treatments accelerated remyelination, with biotin doses providing partial recovery (Fig. 3C.i and D.i), and MgB1 and MgB2 leading to almost complete remyelination at 33 dpi (Fig. 3E.i and F.i) compared to control (Fig. 3A.i), where the high dose of magnesium biotinate showed the most potent effect on remyelination. Considering the typical three-month duration for complete remyelination in rats [66], both biotin and MgB demonstrated a favorable effect, particularly in accelerating the remyelination process. Notably, higher doses of MgB appear to have a more pronounced impact on the acceleration of remyelination. Regarding the routine clinical use of biotin in MS, there are inconclusive results due to safety concerns. However, our results revealed that serum levels of magnesium and biotin were directly correlated with the brain concentrations of Mg and biotin. Additionally, magnesium biotinate treatment led to the normalization of body weight without affecting glucose and lipid metabolism, which might result from the promoting effect of biotin on amino acid metabolism, protein synthesis, and remyelination [67].

Considering that poor oral bioavailability may result in low efficacy and lead to unpredictable side effects, our present data suggest that the combination of magnesium and biotin may exhibit stronger bioavailability than other formulations without producing any side effects. From a translational point of view, when our results were compared with data from recent MS clinical trials, where a high dose of biotin did not seem to be associated with any beneficial effect, the main findings presented here indicate that both low and high amounts of MgB exert significant beneficial impacts on remyelination, inflammation, regeneration, and neurobehavioral scores. As cognitive impairment is the primary factor affecting the quality of life of patients with MS [68], the present data are highly relevant to the clinics of MS, providing insights into the mechanisms of cognitive impairment in patients. From another point of view, biotin-based treatment options have increasingly been recommended as therapeutic tools in degenerative cognitive diseases [69]. Although the combination of magnesium and high-dose biotin emerged as the most effective treatment in reducing injury markers and functional outcomes in this study, it was interesting to observe that magnesium significantly improved almost all parameters when combined with both low and high doses of biotin. This remarkable impact of magnesium, regardless of the biotin dosage, suggests a potent synergistic effect between the two components. Such synergy may be attributed to their complementary mechanisms of action, likely enhancing each other's therapeutic potential, leading to improved overall outcomes. This robust response to the combination therapy highlights the potential of utilizing magnesium as a pivotal adjunct to biotin-based treatments, irrespective of the specific biotin dosage employed. For instance, the biotin-related enzymes PSD-95, GFAP, MMP, OPG, and IL-17A, which showed no change even with higher doses of biotin, responded well to both magnesium and biotin combinations (MgB1 and MgB2). This suggests that combining biotin with magnesium, instead of increasing its dosage, might yield more beneficial effects on the process of remyelination and neurobehavioral outcomes. This finding is consistent with recent prospective MS trials, where applying high-dose biotin as a single agent did not significantly improve disability scores [70].

Taken together, the results of this study show that MgB2 is the most effective formulation for restoring cognitive and neurobehavioral scores and is associated with improved synaptogenesis, remyelination, bioenergetic mechanisms, and alleviated inflammation. Further studies should provide more insights into the dose–response relationships of magnesium and biotin to elucidate the neuroprotective and pro-cognitive efficacy of both substances in detail. Since biotin and magnesium exert no toxic effects, our data may indicate a solid translational therapeutic message in cognitive impairment in both progressive and relapsing–remitting MS forms. In conclusion, increasing the bioavailability of biotin may represent a promising neuroprotective and pro-cognitive therapeutic approach in MS. In contrast, a possible mechanistic link between increased remyelination and bioenergetic enzymes may be of critical importance, a fact of which researchers should be aware.

## Supplementary information

Below is the link to the electronic supplementary material.

Supplementary file (DOCX 5.88 KB)

## Data Availability

The data will be made available at reasonable request.
