# Supplementary material for: Dose-Dependent Effect of a New Biotin Compound in Hippocampal Remyelination in Rats
Source: Mol Neurobiol. 2025 Jan 16;62(5):6503–20. doi: 10.1007/s12035-025-04686-y (PMC11953097; doi:10.1007/s12035-025-04686-y)
Supplement: Supplementary file 1 — Supplementary file (DOCX 5.88 KB) [file 12035_2025_4686_MOESM1_ESM.docx]

**SUPPLEMENTARY INFORMATION**

**Dose-Dependent Role of a New Biotin Compounds in Hippocampal Demyelination in Rats**

Burak Yulug^1^, Ertugrul Kilic^2^, Cemal Orhan^3^, Besir Er^4^, Mehmet Tuzcu^4^, Ibrahim Hanifi Ozercan^5^, Nurhan Sahin^3^, Sinan Canpolat^6^, James Komorowski^7^, Sara Perez Ojalvo^7^, Sarah Sylla^7^, Seyda Cankaya^1^, Kazim Sahin^3,^*

^1^Alaaddin Keykubat University, School of Medicine, Department of Neurology, Alanya, Turkey.

^2^Istanbul Medipol University, Department of Physiology, Istanbul, 34810, Turkey.

^3^Department of Nutrition, Faculty of Veterinary Medicine, Firat University, Elazig, Turkey

^4^Department of Biology, Faculty of Science, Firat University, Elazig, Turkey

^5^Department of Pathology, Faculty of Medicine, Firat University, Elazig, Turkey

^6^Department of Physiology, Faculty of Medicine, Firat University, Elazig, Turkey

^7^Research and Development, Nutrition 21, Harrison, NY 10577, USA

Correspondence: Kazim Sahin, DVM, Ph.D., F.A.C.N. Member of The Turkish Academy of Sciences, Professor of Nutrition Veterinary Faculty, Firat University 23119 Elazig, Turkey. Phone: +904242370000/3938

Email: nsahinkm@yahoo.com, ksahin@firat.edu.tr

(a) IL-6


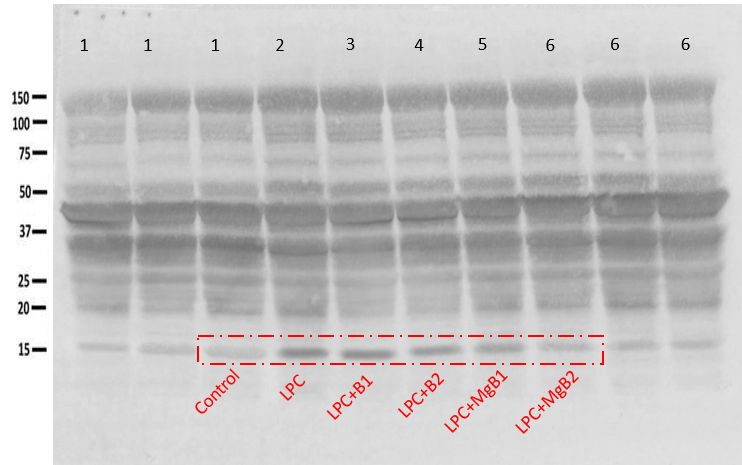


(b) IL-17


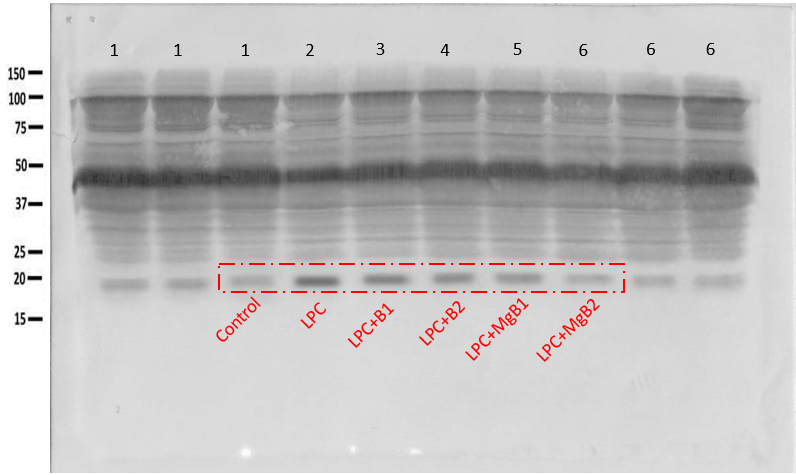


(c) TNF-α


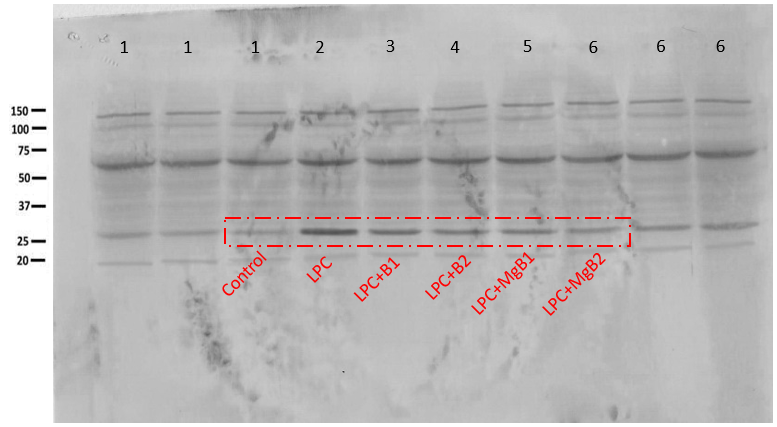


(d) NF-ĸB


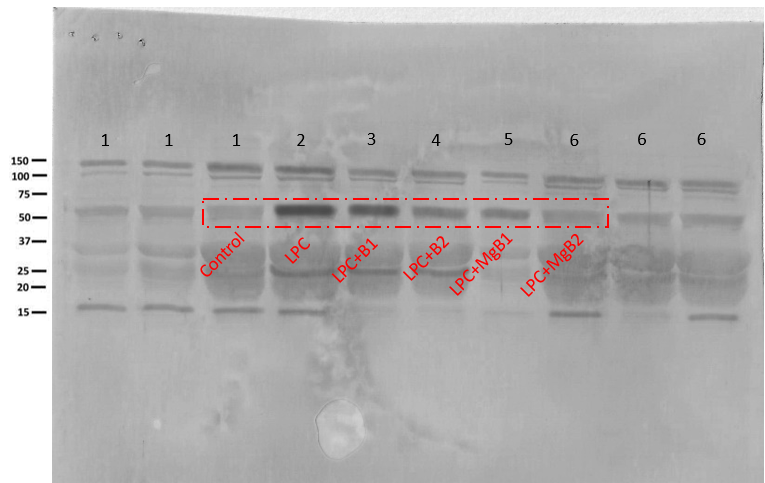


(e) CCL-3


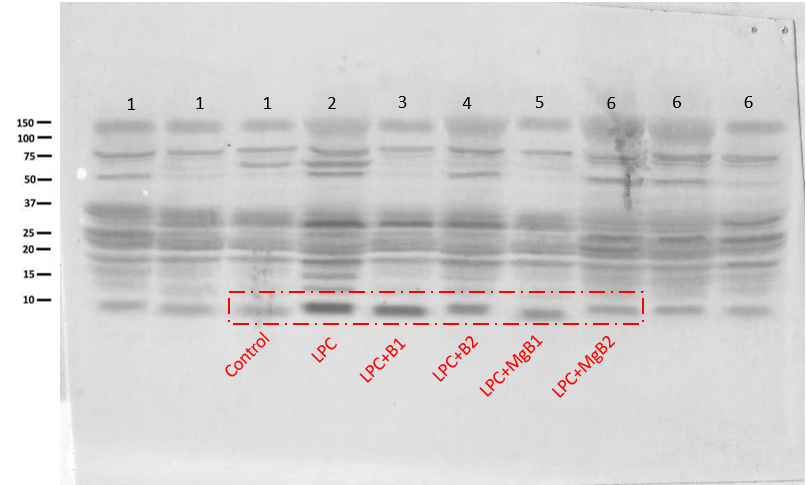


(f) CCL-5


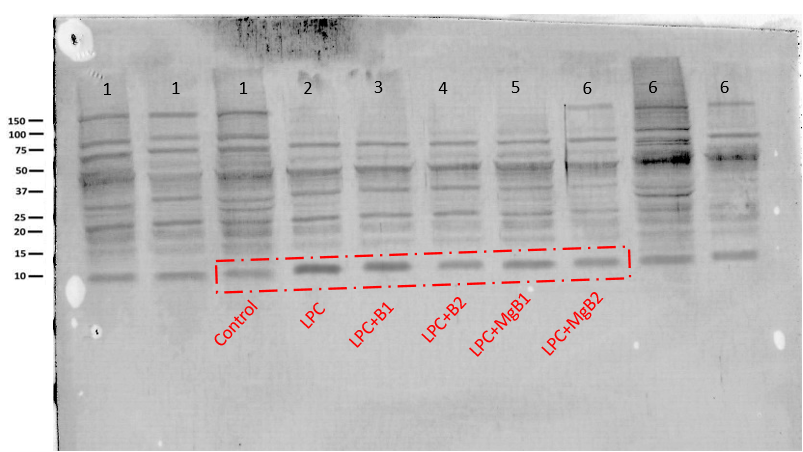


(g) CXCL-16


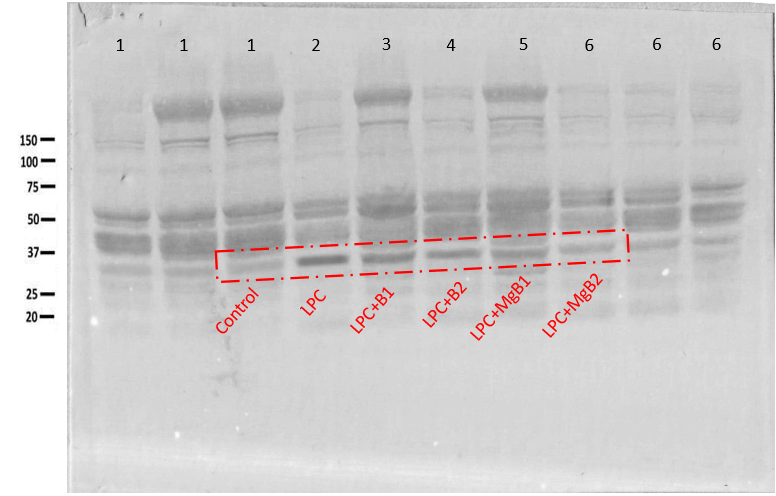


(h) OPG


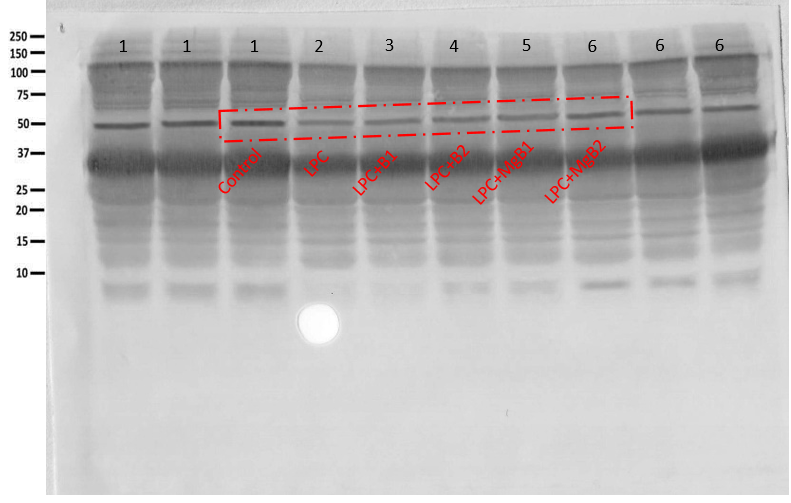


(i) MMP-9


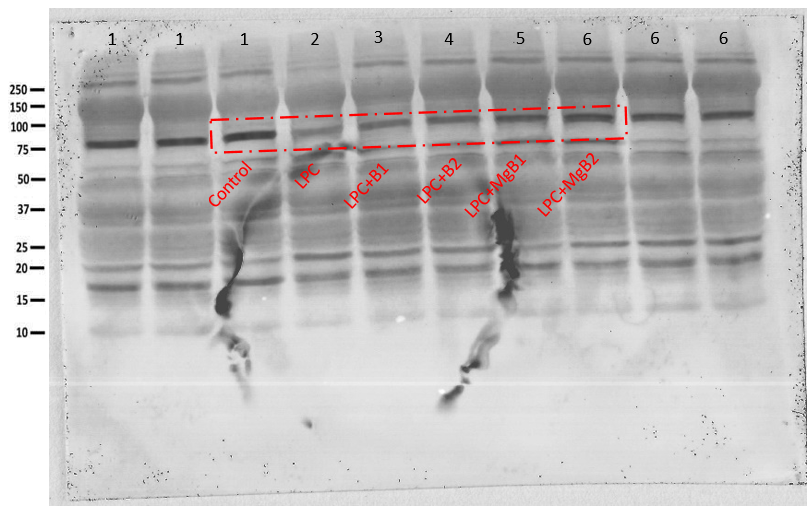


(j) β-Actin (a, b, c)


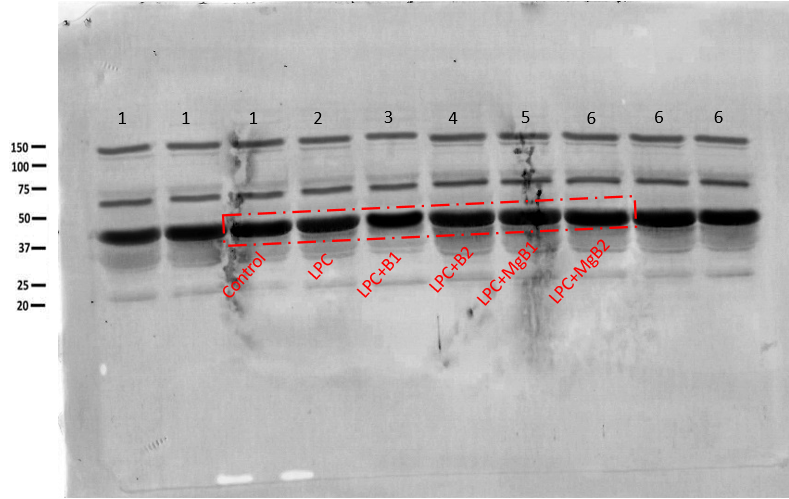


(k) β-Actin (d,e,f)


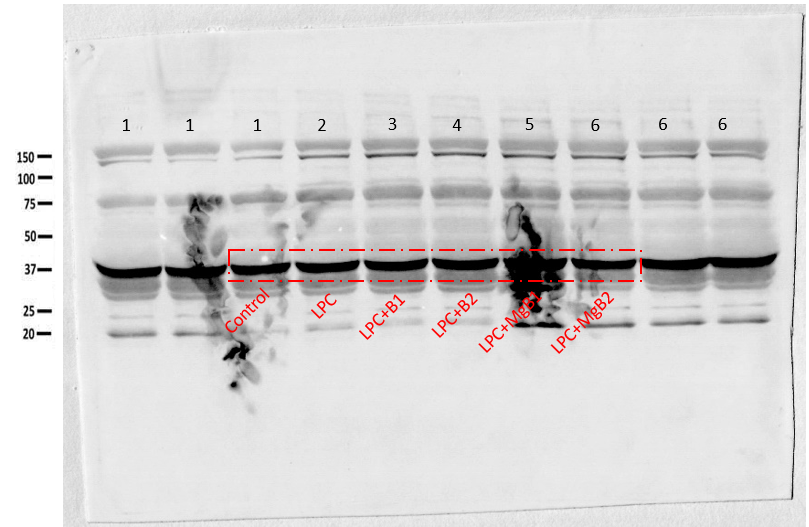


(l) β-Actin (g,h,i)


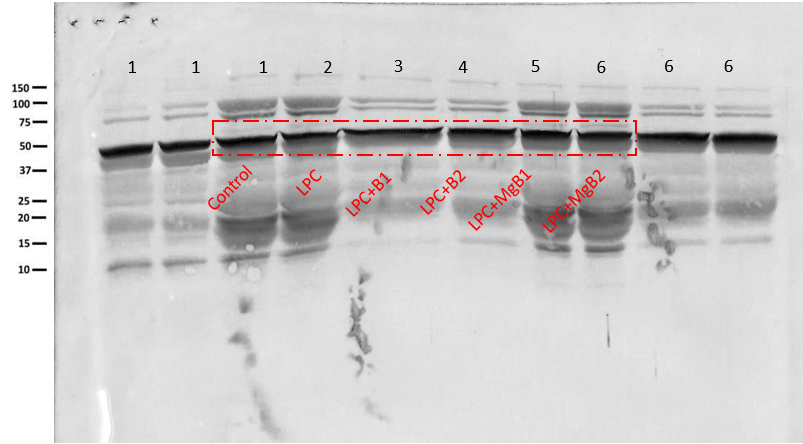


**Supplementary Figure 1.** Full immunoblots related to Fig. 3 on brain tissue of rats; (a) interleukin 6 (IL-6), (b) interleukin 17 (IL-17), (c) tumor necrosis factor alpha (TNF-α), (d) nuclear factor kappa light chain enhancer of activated B cells (NF-κB), (e) chemokine (C-C motif) ligand 3 (CCL-3), (f) chemokine (C-C motif) ligand 5 (CCL-5), (g) chemokine (C-X-C motif) ligand 16 (CXCL-16), (h) osteoprotegerin (OPG), (i) matrix metallopeptidase 9 (MMP-9), (j) β-Actin (a, b, c), (k) β-Actin (d, e, f). and (l) β-Actin (g, h, i). Each immunoblot is a representative of three independent experiments. Results shown in Fig. 3 are delineated by red dotted rectangles. MW (in kDa) are indicated.

(a) ACC-1


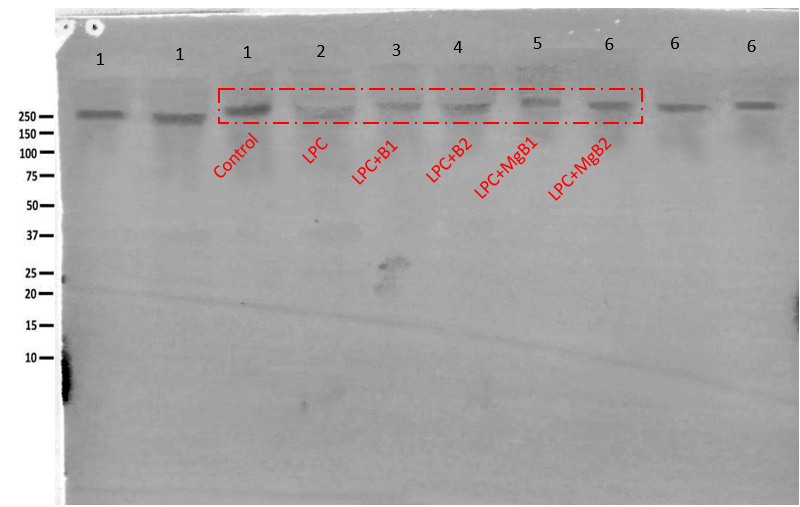


(b) ACC-2


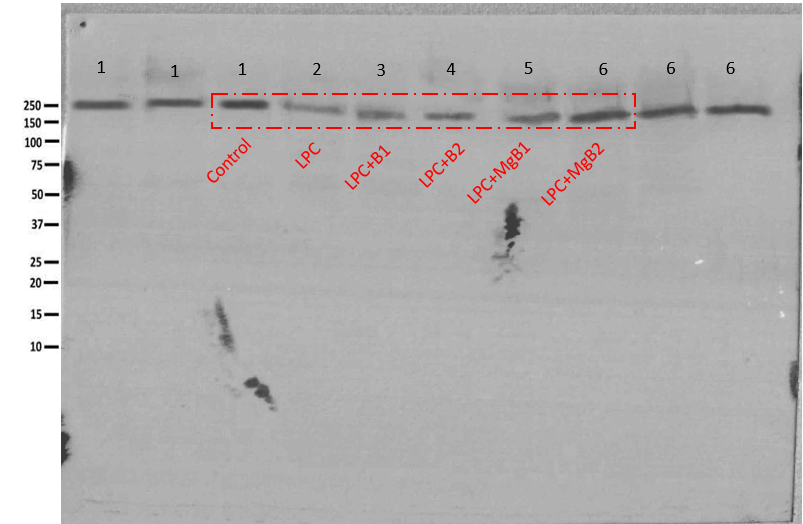


(c) PC


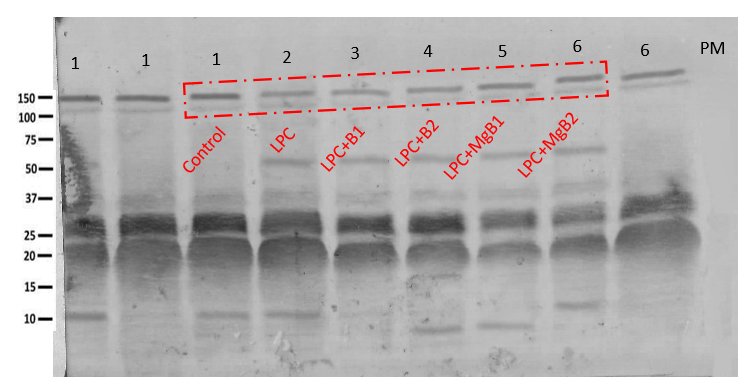


(d) PCC


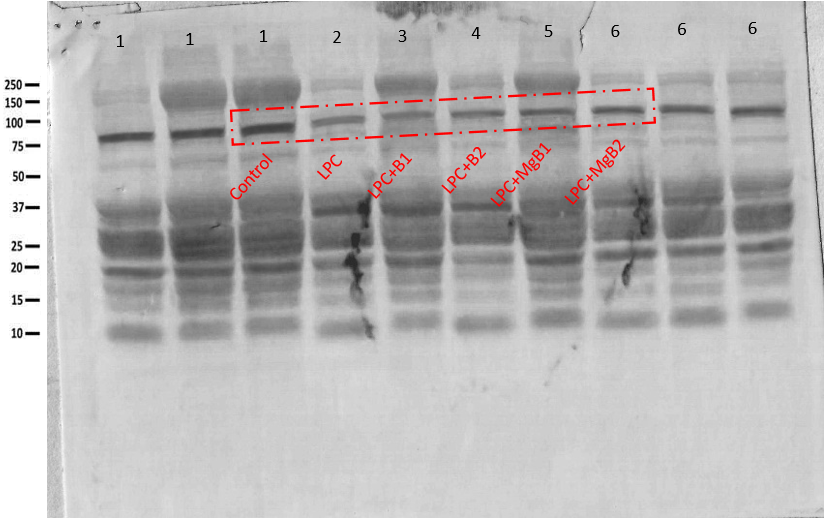


(e) MCC


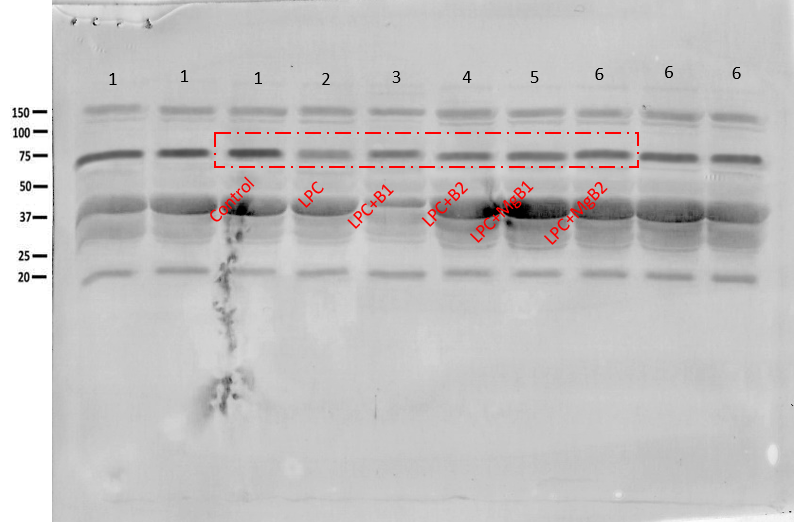


(f) β-Actin


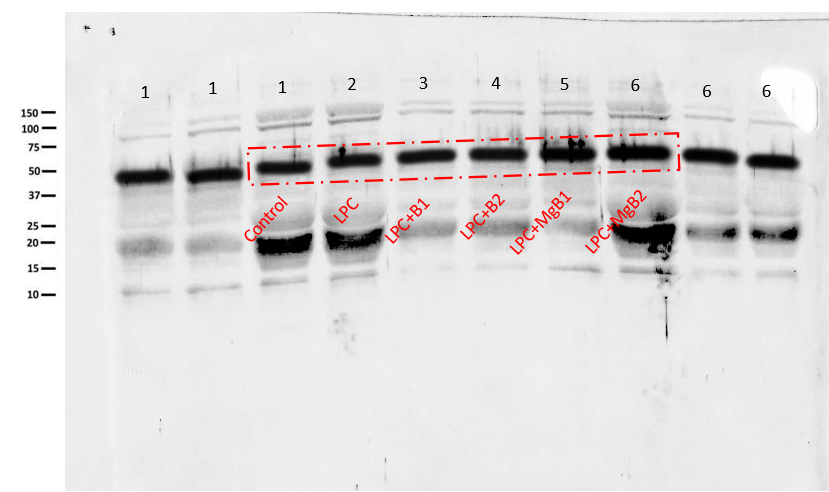


**Supplementary Figure 2.** Full immunoblots related to Fig. 4 on brain tissue of rats; (a) acetyl CoA carboxylase 1 (ACC-1), (b) acetyl CoA carboxylase 2 (ACC-2), (c) pyruvate carboxylase (PC), (d) propionyl-CoA carboxylase (PCC), (e) 3-methylcrotonyl-CoA carboxylase (MCC), and (f) β-Actin. Each immunoblot is a representative of three independent experiments. Results shown in Fig. 4 are delineated by red dotted rectangles. MW (in kDa) are indicated.

(a) BDNF


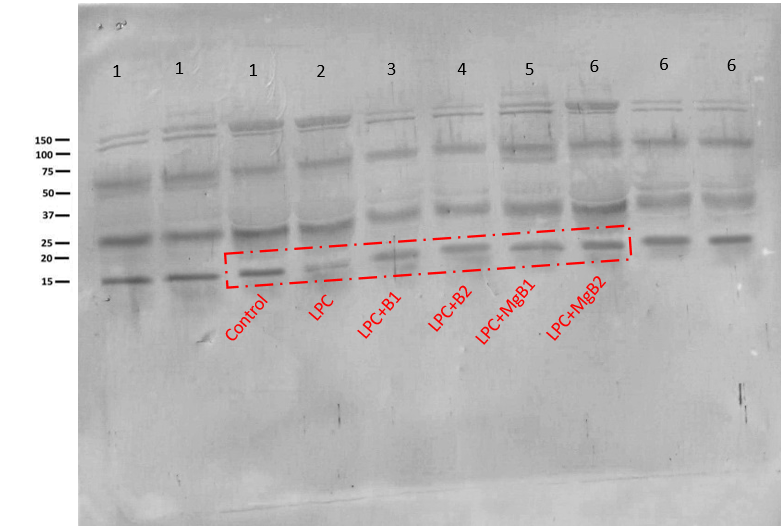


(b) GAP-43


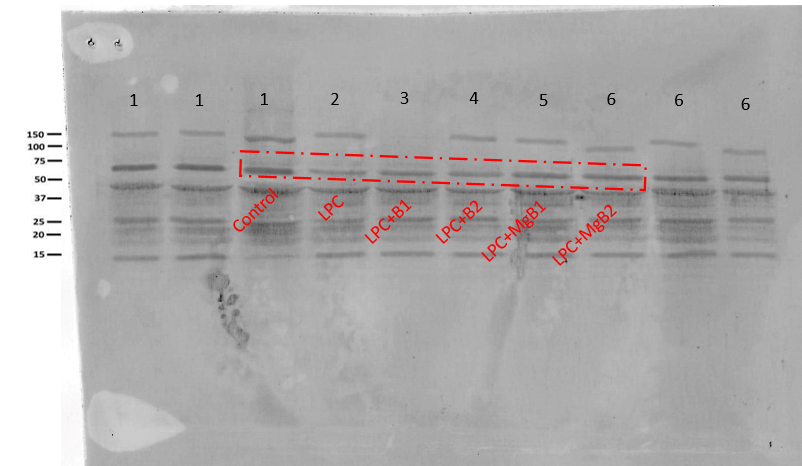


(c) GFAP


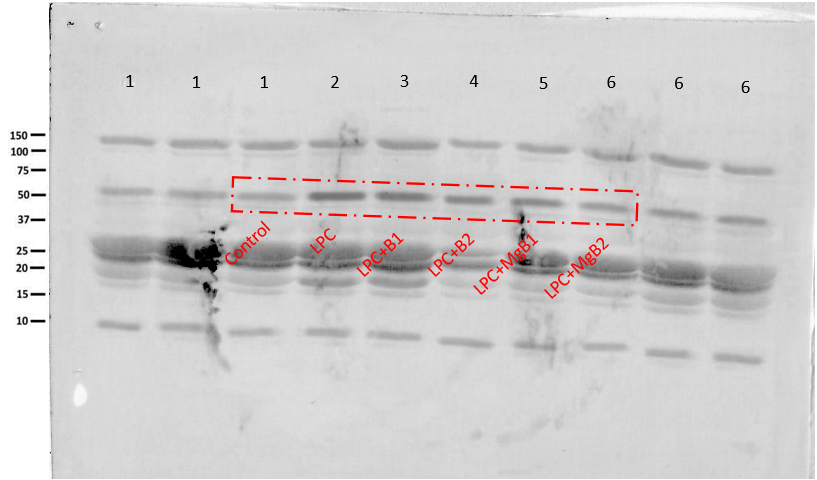


(d) ICAM-1


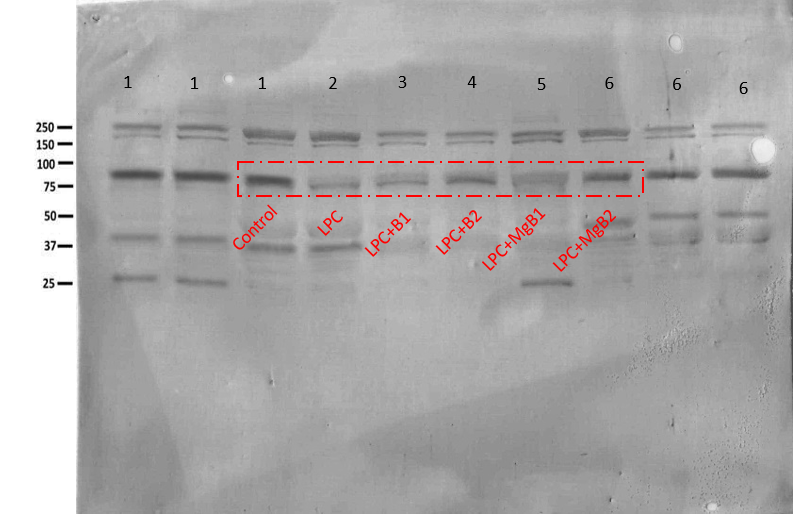


(e) β-Actin


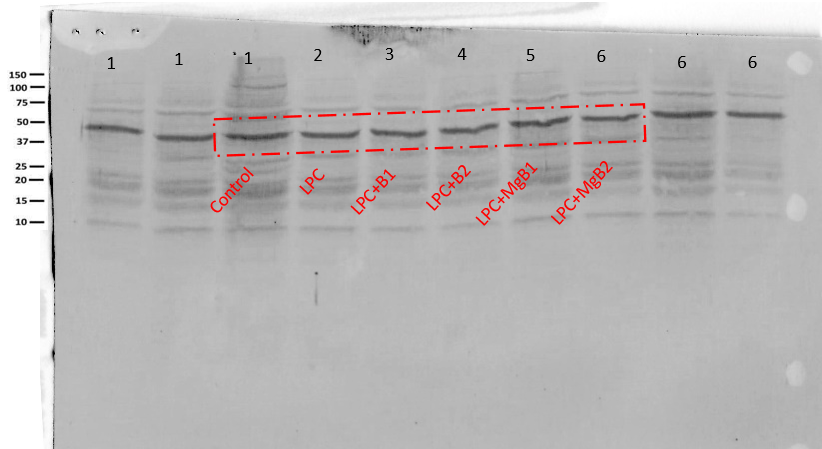


**Supplementary Figure 3.** Full immunoblots related to Fig. 5 on brain tissue of rats; (a) on brain-derived neurotrophic factor (BDNF), (b) growth-associated protein (GAP-43), (c) glial fibrillary acidic protein (GFAP), (d) intercellular adhesion molecule 1 (ICAM-1), and (e) β-Actin. Each immunoblot is a representative of three independent experiments. Results shown in Fig. 5 are delineated by red dotted rectangles. MW (in kDa) are indicated.

(a) Synapsin-I


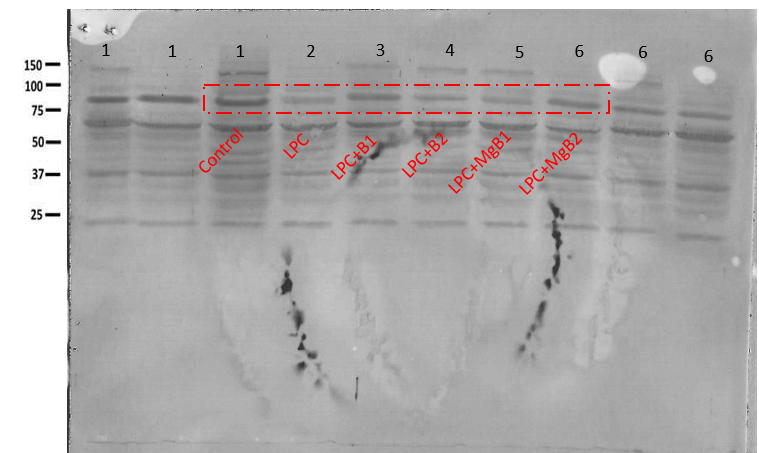


(b) PSD-93


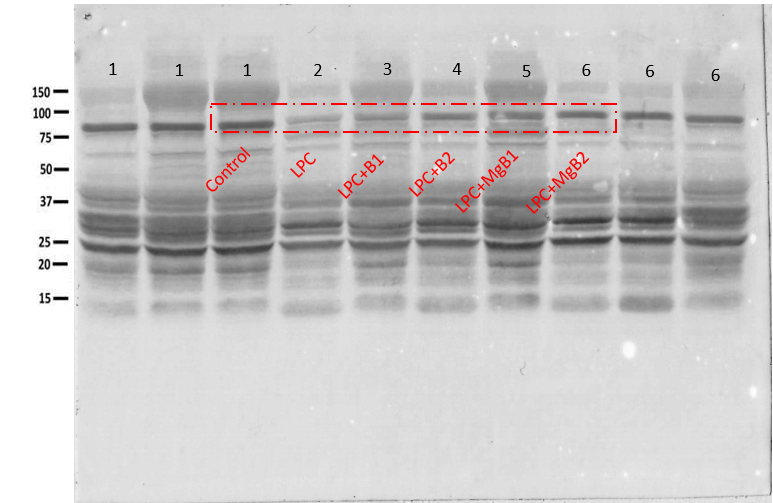


(c) PSD-95


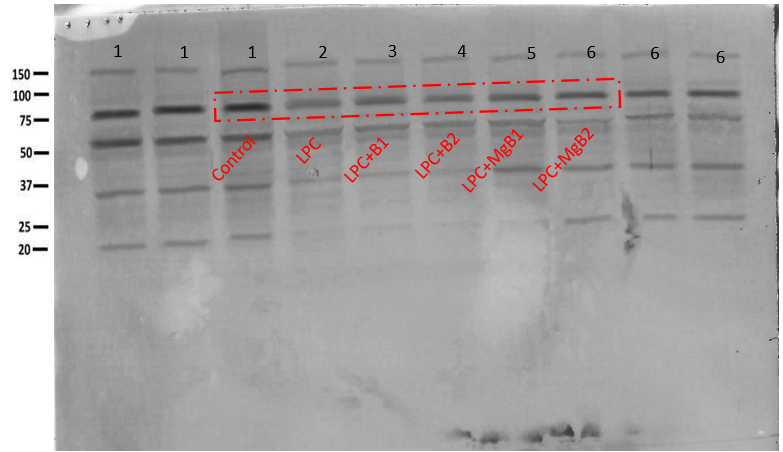


(d) β-Actin


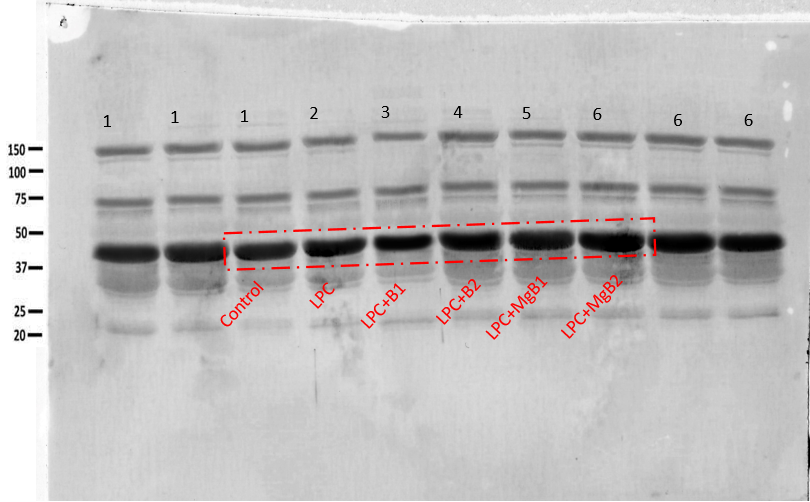


**Supplementary Figure 4.** Full immunoblots related to Fig. 6 on brain tissue of rats; (a) Synapsin-I, (b) postsynaptic density protein 93 (PSD-93), (c) postsynaptic density protein 95 (PSD-95), and (d) β-Actin. Each immunoblot is a representative of three independent experiments. Results shown in Fig. 6 are delineated by red dotted rectangles. MW (in kDa) are indicated.
